# Identifying Etiologically Distinct Sub-Types of Cancer: A Demonstration Project Involving Breast Cancer

**DOI:** 10.1002/cam4.456

**Published:** 2015-05-13

**Authors:** Colin B Begg, Irene Orlow, Emily C Zabor, Arshi Arora, Ajay Sharma, Venkatraman E Seshan, Jonine L Bernstein

**Affiliations:** Department of Epidemiology and Biostatistics, Memorial Sloan Kettering Cancer CenterNew York City, New York

**Keywords:** Double primaries, etiologic heterogeneity, risk prediction, tumor subtypes

## Abstract

With the advent of increasingly detailed molecular portraits of tumor specimens, much attention has been directed toward identifying clinically distinct subtypes of cancer. Subtyping of tumors can also be accomplished with the goal of identifying distinct etiologies. We demonstrate the use of new methodologies to identify genes that distinguish etiologically heterogeneous subtypes of breast cancer using data from the case–control Cancer and Steroid Hormone Study. Tumor specimens were evaluated using a breast cancer expression panel of 196 genes. Using a statistical measure that distinguishes the degree of etiologic heterogeneity in tumor subtypes, each gene is ranked on the basis of its ability to distinguish etiologically distinct subtypes. This is accomplished independently using case–control comparisons and by examining the concordance odds ratios in double primaries. The estrogen receptor gene, and others in this pathway with expression levels that correlated strongly with estrogen receptor levels, demonstrate high degrees of etiologic heterogeneity in both methods. Our results are consistent with a growing literature that confirms the distinct etiologies of breast cancers classified on the basis of estrogen receptor expression levels. This proof-of-principle project demonstrates the viability of new strategies to identify genomic features that distinguish subtypes of cancer from an etiologic perspective.

## Introduction

It has long been recognized that cancers defined by disease site are not homogeneous disease entities. Indeed since the advent of the genomics era many investigations have been undertaken to identify disease subtypes that are clinically distinct 1–4. Research on the topic of etiologic heterogeneity has usually involved the investigation of distinct risk factor profiles of predetermined subtypes, such as studies that have demonstrated the distinctive relationship of smoking history on lung cancer histologic types 5,6, and the considerable body of work that has examined differences in the risk factor profiles of breast cancers defined by hormonal disease markers 7–18. In this article, we seek to demonstrate the application of two parallel methodological strategies for exploring tumor characteristics as a means to identify those characteristics that best define groups of cases with distinctive etiologies.

The investigation of etiologic heterogeneity from the perspective of defining subtypes is not immediately straightforward for several reasons. On one hand, cancer risk may be influenced by many factors. On the other hand, the somatic characteristics of tumors may be represented in many different ways, and on the basis of large numbers of markers. We thus need an organizing framework for simultaneously investigating numerous candidate groups of subtypes while distinguishing promising candidates from unpromising ones. To address this problem, in previous work our group has proposed a scalar measure of etiologic heterogeneity that captures the distinctiveness of risk factor profiles for a set of candidate subtypes 19. This can be used to rank subtyping options and identify the ones that appear most promising on the basis of known risk factors using data from case–control studies. We have also shown that a very similar measure can be derived using the concordance odds ratio of the subtypes in double primary cancers 20.

The goal of this article is to report a demonstration of these two strategies using data from a well-characterized case–control study of breast cancer in which available tumor tissue was used to conduct expression profiling of a panel of 196 genes linked with breast cancer. We first contrast the risk profiles of the cases and controls in subtypes created on the basis of expression levels of each of the genes in turn. This allows us to rank the genes on the extent to which they define etiologically distinct subtypes. In parallel, we evaluated tumor tissue from both tumors of women with contralateral breast cancer using the same expression panel, and used the odds ratios from the cross-classifications of the tumors for each gene in turn to rank the genes on the extent to which they define etiologically distinct subtypes. The two rankings are then compared to identify genes that exhibit a high rank by both methods.

## Material and Methods

### Data

We use data from the Cancer and Steroid Hormone (CASH) Study, augmented with additional molecular profiling of tumor specimens. The CASH Study was led by the Centers for Disease Control in the early 1980s. The investigators made use of the Surveillance, Epidemiology and End Results (SEER) registries for identification of incident cases of breast cancer 21,22. The cases were women aged 20–56 with invasive primary breast cancer diagnosed between 1980 and 1982 in eight SEER registries. Unaffected frequency-matched controls were ascertained through random digit dialing in the geographic areas served by the study registries. All participants were interviewed at the time the study was conducted to determine known and suspected risk factors including age at diagnosis, age at menarche, nulliparity, number of children, age at first birth, months of breastfeeding, body mass index (BMI), menopausal status, age at menopause, race, prior benign breast disease, and family history of breast cancer. These characteristics are described in Table1 for the subset of women for whom tumor tissue was available and for their frequency-matched controls. For modeling purposes, we used imputations for items such as age at first birth for nulliparous women following the guidelines advocated by Thompson 23. In an earlier study, tumor tissue was evaluated using immunohistochemistry for the estrogen receptor alpha (ESR1), progesterone receptor (PGR), human epidermal growth factor receptor (ERBB2), and TP53 12.

**Table 1:** Risk factor distributions

Risk factor	Controls (*n* = 2990)	Cases (*n* = 551)
Age (range)	47 (20,55)	47 (24,55)
White race (%)	87%	93%
Premenopausal BMI, median (range)	23 (16,55)	23 (16,44)
Postmenopausal BMI, median (range)	24 (16,62)	24 (17,35)
Family history of breast cancer	7%	13%
Prior benign breast disease	12%	18%
Age at menarche, median (range)	13 (8,20)	12 (8,18)
Nulliparity	14%	15%
Number of children, median (range)	3 (1,13)	3 (1,9)
Age at first birth, median (range)	23 (11,43)	23 (13,40)
Months of breastfeeding, median (range)	1 (0,168)	1 (0.98)
Postmenopausal	40%	37%
Age at menopause, median (range)	42 (21.53)	42 (23,53)
Estrogen receptor positive by IHC^1^	NA	57%
Progesterone receptor positive by IHC^1^	NA	51%
*ERBB2* (HER2) positive by IHC^1^	NA	21%

BMI, body mass index; IHC, immunohistochemistry; *ERBB2*, human epidermal growth factor receptor.

1 33 cases are missing immunohistochemistry data.

### RNA extraction and molecular profiling

Total RNA was isolated from precut 4 *μ*m formalin-fixed paraffin-embedded sections with the RNeasy FFPE kit (Qiagen) using the manufacturer's recommendations. The RNA quantities and A260/280 ratios were determined with a Nanodrop 2000 (Thermo Scientific/NanoDrop products, Wilmington, DE, USA) and the A260/280 ratios had an average value of 1.79 (range 1.28–2.26).

Tumor tissues were profiled using a targeted breast cancer expression panel, and the results were used to determine if expression of genes other than *ESR1*, *PGR*, and *ERBB2* contain relevant signals that can improve our ability to define etiologically distinct subtypes. Gene expression analysis was performed using the NanoString nCounter^R^ Virtual Breast Cancer—Estrogen panel 24. This measures expression levels of 196 human genes known to be differentially expressed in breast cancers or relevant to estrogen receptor signaling. The genes encompass other breast cancer-related pathways including the following: apoptosis; epidermal growth factor receptor; Fas cell surface death receptor; interleukin; androgen/estrogen/progesterone biosynthesis; transforming growth factor beta signaling; p53; *RAS-RAF-MEK-ERK*. A full list of genes in the panel is provided in Table S1. The nCounter^TM^ (Nanostring, Seattle, WA, USA) expression assays were run using 500 ng of RNA following manufacturer's recommendations. Briefly, hybridizations were carried out at 65°C for 18 h on a thermocycler. After the posthybridization processing in an nCounter Prep station samples were scanned using 600 fields of view on an nCounter Digital Analyzer (NanoString Technologies, Seattle, WA). Raw transcript counts were subtracted from background (negative input control).

### Expression data normalization, technical reproducibility, and robustness

We used the nSolver software analysis. The mean of each of the positive controls for each sample was calculated with the software tool to estimate the overall assay efficiency. Counts were normalized for all target RNAs in all samples based on the positive control RNA to account for differences in hybridization efficiency and posthybridization processing, including purification and immobilization of complexes (samples <0.3 or >3 were flagged). Subsequently the mRNA content normalization was performed using a panel of six reference genes (*CLTC, GAPDH, GUSB, HPRT1, PGK1, TUBB*). For each sample, we calculated the ratio of endogenous counts to the average of endogenous counts across all samples. Samples with ratios below 0.10 or above 10 were flagged and repeated.

### Analytic strategy

The goal of the analysis is to identify tumor subtypes that are etiologically distinct. In the previous work, we have outlined how novel clustering methods can be used to identify subtypes 19. However in this demonstration project where tissue was available from only 44 double primaries, we have limited the scope of our analysis to identifying genes individually that demonstrate evidence that they can distinguish etiologically heterogeneous subtypes. Specifically, the use of a quantitative measure of etiologic heterogeneity, denoted *D*, allows us to rank genes to determine the extent to which the individual genes are capable of identifying etiologically distinct subtypes. For each gene, we calculate *D* by contrasting the distinctive risk profiles for subtypes classified at the median into high (H) versus low (L) expressions of the gene. The rationale for using *D* for this purpose was explained in detail in our earlier publication 19. If individuals with a high risk of H tumors also tend to have a high risk of L tumors and vice versa then the risk profiles are closely aligned and the subtypes possess low etiologic heterogeneity. Conversely, if the risks of H tumors are unrelated to the risks of L tumors then the tumors have distinct, that is, independent, risk profiles, and are thus etiologically heterogeneous. *D* is defined as follows:

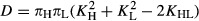
1where 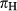 and 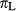 are the relative frequencies of the two subtypes, 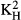 is the coefficient of variation in the risks of type H cancer in the population, 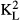 is the coefficient of variation in the risks of type L cancer in the population, and 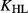 is the coefficient of covariation in these risk profiles. Note that *D* is negatively related to the correlation in risk profiles. All of these terms can be estimated directly based on observable risk factors using data from the case–control study. Specifically, we perform a polytomous logistic regression comparing cases in subtypes H and L with the common control group. We then use the estimated parameters from this model to predict the risks of subtype H cancers and subtype L cancers for all control subjects, and use these risks to calculate directly the terms 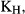 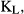 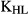 and thus *D*. We evaluate the statistical significance of the heterogeneity signal by randomly allocating H/L labels to the cases, conducting the polytomous regression, calculating individual risks and the measure *D*, and repeating this process a large number of times (100,000) to obtain a reference distribution. The *P*-value is the proportion of these permuted values of *D* that are at least as large as the observed value.

We also evaluate the etiologic heterogeneity explained by each of the genes using a completely independent methodology. In this approach, we focus solely on the co-occurrence of subtypes in both tumors among cases with double primaries. That is, we cross-classify the pairs of double primaries on the basis of high versus low expression of the gene of interest and calculate the odds ratio. In an earlier paper 20, we showed that the logarithm of this odds ratio (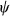), weighted as above by 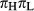 is a first-order approximation to the term *D* in (1) above. Specifically

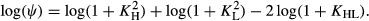


Based on this similarity, we use the following as our corresponding heterogeneity measure:

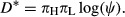
13

We use this to rank the genes on the basis of their etiologic heterogeneities as before. We note that in this method all risk factors for breast cancer influence the heterogeneity measure, regardless of whether they are known or unknown. By contrast, in the previous (case–control) method the heterogeneity measure reflects only the risk factors that are employed in the analysis. Nonetheless we regard it as confirmatory if a gene has a high rank on both methods.

Since for each of these analyses we are ranking and testing a large number of genes we must account for the possibility of false discovery. This was evaluated by calculating the false discovery rate 25 and identifying those genes with a sufficiently high observed value of *D* that the false discovery rate adjusted for multiple testing is less than 5%. Comparisons of the odds ratios for individual risk factors from the polytomous logistic regression analyses allow us to identify the risk factors that have the most distinctive relative risks for the different subtypes.

## Results

Of the 738 tested RNA samples 31 failed various quality measures, resulting in 707 evaluable samples of which 657 were first primaries and 50 were second primaries. Fourteen of the 657 single primary samples were technical duplicates and six of the 50 sec primary samples did not have a matching pair after exclusions due to failures. In addition, 92 cases were missing key risk factor data resulting in a final sample size for analysis of 551 incident cases of breast cancer with available expression and risk factor data. Control data on breast cancer risk factors were available from 2990 population controls from the four CASH Study centers that contributed case tumor tissue. We evaluated the gene expression results by comparing the expression levels obtained with the Nanostring nCounter Virtual Breast Cancer for both the estrogen (*ESR1*) and *PGR* genes with immunohistochemistry results from an earlier study 19. The ROC curves in Figure S1 demonstrate close concordances, in line with Du et al. 26.

We ranked the genes on the basis of the heterogeneity measure for both the case–control method and the double primaries method, as outlined in Methods. Results for the top 20 genes (10% of the 196 genes evaluated) for each method are displayed in Table2. (Results for all 196 genes in the panel are available in Table S2). For the case–control analyses, the top nine genes remain significant at the 5% level after adjusting for multiple testing. For the double primary analyses only the top ranked gene (*KRT19*) survives the multiple testing adjustment. However, it must be recognized that the double primary analysis is based on only 44 cases and so it has limited power. Because of the power limitation we focus attention on any highly ranked genes identified in Table2 rather than those that are statistically significant after adjustment for multiple testing.

**Table 2:** Top ranked genes on basis of etiologic heterogeneity

Double primary analysis				Case–control analysis		
Gene^1^	OR^2^	D^*^	*P*-value	Gene^1^	*D*	*P*-value
***KRT19***	13.8	0.66	<0.001^3^	*GATA3*	0.11	<0.001^3^
*HSD17B1*	8.2	0.52	0.002	***ESR1***	0.11	<0.001^3^
*TOP2A*	7.3	0.50	0.005	***IL6ST***	0.10	<0.001^3^
***IL6ST***	6.4	0.46	0.006	*TFF1*	0.10	<0.001^3^
*PRMT5*	6.3	0.46	0.007	*BCL2*	0.10	<0.001^3^
*ID2*	6.3	0.46	0.016	*HMGB1*	0.10	<0.001^3^
*MK167*	5.5	0.43	0.021	*TFF3*	0.09	<0.001^3^
*ATR*	5.5	0.42	0.015	***NRIP1***	0.09	<0.001^3^
***ESR1***	5.5	0.42	0.015	*BCL2L2*	0.09	<0.001^3^
***PGR***	5.5	0.42	0.015	*SULT2A1*	0.09	<0.001
*AZGP1*	5.4	0.42	0.015	***PGR***	0.08	<0.001
*NME1*	4.5	0.38	0.019	*GTF2F1*	0.08	<0.001
*MT3*	4.4	0.37	0.032	*ERCC3*	0.08	<0.001
*MUC1*	4.4	0.37	0.032	*NFYB*	0.08	<0.001
*HSPB1*	4.4	0.37	0.040	*JUN*	0.08	<0.001
***NRIP1***	3.8	0.33	0.053	*SLC7A5*	0.08	0.001
*SERPINA3*	3.8	0.33	0.040	*GABRP*	0.08	0.001
*RPL27*	3.7	0.32	0.065	***KRT19***	0.08	0.001
*CHEK1*	3.6	0.32	0.063	*F3*	0.08	0.002
*CDH1*	3.4	0.31	0.066	*RAD50*	0.08	0.002

OR, odds ratio; *ESR1*, estrogen receptor alpha; *PGR*, progesterone receptor.

1 Genes in boldface are represented in both lists.

2 Odds ratio.

3 These comparisons are significant at the 5% level after adjustment for multiple comparisons.

The estrogen receptor gene (*ESR1*) appears among the top ranked genes in both analyses, confirming the extensive evidence in the literature that breast cancers classified on the basis of expression levels of this gene have distinctive etiologies. Of the remaining four genes that also were highly ranked by both analyses, some have expression profiles that are quite strongly correlated with *ESR1* expression: *IL6ST* (correlation with *ESR1* is 0.75); *PGR* (correlation with *ESR1* is 0.70); *NRIP1* (correlation with *ESR1* is 0.69). Only *KRT19* has a somewhat more modest correlation with *ESR1* (0.49). These results reflect the fact that the gene panel contained many genes related to estrogen receptor signaling, and the fact that three of the other four genes have very strong correlation with *ESR1* confirms the dominance of this pathway in distinguishing etiologically distinct breast cancers.

We examined for each risk factor the relative risks of the individual subtypes based on each gene and identified those that are significantly different for the two subtypes. Since four of the five selected genes are highly correlated, the relative risk patterns are quite similar for the analyses based on each of these genes. As a representative example we display in Table3 the results for the overall top ranked gene, *IL6ST*. In the table, the overall odds ratios using all cases are contrasted with the subtype-specific odds ratios. Thus, for example, the high expression subtype is characterized by older age at diagnosis, lower premenopausal BMI, etc. The results suggest that the risk factors that are primarily driving the etiologic heterogeneity are age at diagnosis, previous benign breast disease, and menopausal status. In Table4, we present the corresponding results for *KRT19* expression, the selected gene least highly correlated with *ESR1*, but the observed associations are generally weaker and no additional risk factors emerge.

**Table 3:** Odds ratios^1^ for subtypes defined by expression levels of the *IL6ST* gene

Risk factor	All cases	Subtypes		Test for heterogeneity
		High expression	Low expression	
Age at reference (per 10 years)	1.4 (1.2–1.6)	1.8 (1.4–2.2)	1.1 (0.9–1.3)	<0.001
Non-white race	0.7 (0.5–1.0)	0.7 (0.4–1.2)	0.7 (0.4–1.1)	0.86
Premenopausal BMI (per 20 units)	1.2 (0.6–2.1)	0.6 (0.2–1.4)	2.0 (1.0–4.0)	0.03
Postmenopausal BMI (per 20 units)	1.1 (0.5–2.3)	1.0 (0.3–3.1)	1.1 (0.4–2.9)	0.91
Family history of breast cancer	2.0 (1.5–2.6)	2.0 (1.4–3.0)	1.9 (1.3–2.9)	0.87
Prior benign breast disease	1.6 (1.2–2.0)	2.1 (1.5–2.9)	1.1 (0.7–1.6)	0.004
Age at menarche (per 2 years)	0.9 (0.8–1.1)	1.0 (0.8–1.2)	0.9 (0.7–1.0)	0.24
Nulliparous	1.3 (1.0–1.7)	1.7 (1.2–2.4)	1.1 (0.7–1.5)	0.06
Parity	0.9 (0.8–0.9)	0.8 (0.7–1.0)	0.9 (0.8–1.0)	0.48
Age at first birth (per 5 years)	1.1 (0.9–1.2)	1.1 (0.9–1.3)	1.1 (0.9–1.3)	0.99
Months of breastfeeding (per 6 months)	0.9 (0.8–1.0)	1.0 (0.8–1.1)	0.8 (0.7–0.9)	0.04
Postmenopausal	0.7 (0.6–0.9)	0.5 (0.3–0.7)	1.0 (0.7–1.3)	<0.001
Age at menopause (per 5 years)	1.0 (0.9–1.1)	1.0 (0.9–1.2)	1.0 (0.9–1.2)	0.84

BMI, body mass index.

1 Odds ratios and 95% confidence intervals adjusted for all factors in the table.

**Table 4:** Odds ratios^1^ for subtypes defined by expression levels of the *KRT19* gene

Risk factor	All cases	Subtypes		Test for heterogeneity
		High expression	Low expression	
Age at reference (per 10 years)	1.4 (1.2–1.6)	2.0 (1.6–2.4)	1.0 (0.8–1.2)	<0.001
Non-white race	0.7 (0.5–1.0)	0.7 (0.4–1.1)	0.7 (0.4–1.1)	0.80
Premenopausal BMI (per 20 units)	1.2 (0.6–2.1)	0.6 (0.2–1.4)	2.0 (1.0–4.0)	0.02
Postmenopausal BMI (per 20 units)	1.1 (0.5–2.3)	1.5 (0.6–4.0)	0.7 (0.3–2.2)	0.33
Family history of breast cancer	2.0 (1.5–2.6)	1.6 (1.1–2.4)	2.4 (1.7–3.5)	0.13
Prior benign breast disease	1.6 (1.2–2.0)	1.8 (1.3–2.5)	1.3 (0.9–1.9)	0.30
Age at menarche (per 2 years)	0.9 (0.8–1.1)	0.9 (0.8–1.1)	0.9 (0.8–1.1)	0.82
Nulliparous	1.3 (1.0–1.7)	1.3 (0.9–1.9)	1.3 (0.9–1.9)	0.90
Parity	0.9 (0.8–0.9)	0.8 (0.7–0.9)	0.9 (0.8–1.0)	0.09
Age at first birth (per 5 years)	1.1 (0.9–1.2)	1.0 (0.9–1.2)	1.1 (0.9–1.3)	0.81
Months of breastfeeding (per 6 months)	0.9 (0.8–1.0)	0.9 (0.8–1.0)	0.9 (0.8–1.0)	0.93
Postmenopausal	0.7 (0.6–0.9)	0.6 (0.4–0.8)	0.9 (0.6–1.2)	0.04
Age at menopause (per 5 years)	1.0 (0.9–1.1)	1.1 (0.9–1.3)	1.0 (0.8–1.2)	0.44

BMI, body mass index.

1 Odds ratios and 95% confidence intervals adjusted for all factors in the table.

## Discussion

Our purpose was to demonstrate an approach for investigating etiologic heterogeneity. Because of the availability of tissue samples from the case–control study in addition to available tissues from cases with double primary breast cancers, we were able to investigate two entirely different and totally independent strategies. This provides greater credibility regarding genes for which strong heterogeneity signals were observed by both methods. Our major observation is that the estrogen receptor gene and a few other strongly correlated genes demonstrated the strongest consistent signals using both approaches. This is a reassuring result from a methodological perspective in that it supports a body of evidence that has emerged over the past decade that expression of the estrogen receptor gene is the strongest known factor that distinguishes etiologic subtypes of breast cancer. The *KRT19* gene demonstrated the highest heterogeneity signal in the double primary analysis and also ranked highly in the case–control analysis. This gene is a member of the type 1 keratin family and has been associated with survival and dormancy of breast cancer cells as well as with migration and invasion 27–29. Our analyses also suggest a subset of known breast cancer risk factors that appear to most clearly distinguish the subtypes.

Previous studies have demonstrated the importance of estrogen expression in classifying cases into clinically distinct subtypes. The pioneering study by Sørlie et al. 30. identified four subtypes from a clustering analysis of genome-wide expression arrays that can be approximated by expression levels of *ESR1, PGR*, and *ERBB2*. Much clinical and epidemiological research over the past decade has focused on clinical and epidemiological distinctions between these subtypes. A more comprehensive recent study of over 2000 cases using an integrated analysis of expression and copy number profiling suggested a considerably more refined architecture, with 10 distinct subtypes 31. We recognize that our study is neither large enough with respect to numbers of cases, nor with respect to the extensiveness of the gene expression profiling, to produce definitive findings with respect to subtype identification. Consequently we have focused attention on demonstrating our novel analytic strategies in the context of identifying individual genes that contribute strongly to distinctive etiologies. Future studies with considerably larger numbers of cases are needed to approach the task of clustering cases into subtypes based on the contributions of combinations of many such genes.

There is strong rationale for investigating etiologic heterogeneity. If a disease group is really a mixture of cases with distinct etiologies then the resulting signal from any risk factor that is restricted to one of the subtypes will be diluted in the aggregate disease group, greatly reducing the power to detect such signals 32. Thus, identification of etiologically distinct subtypes can improve substantially the power to detect unknown risk factors. The study of double primary cancers is an especially useful strategy from this perspective in that the subtypes such an analysis reveals are influenced by all risk factors, both known and unknown 20. Subtypes identified in this way are optimal for discovering unknown risk factors in future case–control or cohort studies. For this reason double primaries with available tumor tissue from both primaries are an especially valuable resource that can provide unique insights concerning cancer risk. However, accession of tumor tissue from both primaries, especially metachronous primaries, is challenging, and this is reflected in the relatively small number of such cases in our own study. Our analogous strategy for investigating etiologic heterogeneity using case–control studies where the tissues are available for genomic interrogation is more immediately practical in that larger sample sizes are more readily available. These studies rely on information from known risk factors to identify the etiologically distinct subtypes, and one cannot make direct inferences about the chances that the subtypes so identified will segregate also on unknown risk factors. However, it is a reasonable supposition that subtypes that are clearly distinct with respect to established risk factors may also be distinct with respect to at least some of the unknown risk factors.

Our study has a number of limitations. First, the sample sizes are too small for a definitive evaluation of the complex issue we seek to investigate. We set up the study from the perspective of a demonstration project of a novel methodology and we recognize that the results are necessarily speculative. The strategy relies on laboratory evaluation of tumor tissues and these are not typically available in epidemiologic studies. Second, there is no evidence to indicate which of the many genomic profiling options are likely to most clearly identify subtypes with distinctive etiologies. Do somatic DNA events such as mutations, copy number changes, or methylation patterns contain the basic information that distinguishes etiology? Or are phenotypic tumor characteristics such as expression patterns more likely to carry the signal? In constructing our project we thus had very limited evidence to guide our choices regarding molecular profiling. We elected to use an expression panel in large part because previous studies have demonstrated the presence of etiologic heterogeneity based on expression markers, notably based on the estrogen receptor gene 7–18. Also, clinical subtyping strategies have focused primarily on expression profiling, though recent studies have greatly broadened the focus of investigation 3. In short, we elected to study RNA expression because we knew for sure that signals exist. However, we recognize that expression profiling may not be the optimal platform for this purpose. Indeed there is a growing literature supporting the premise that methylation patterns may usefully delineate etiologically distinct subtypes 33–37. Third, the use of expression profiling introduces potential bias into our analyses of double primaries that would not be applicable if we were using genomic platforms that identify only somatic mutations. The expression levels of individual genes may be correlated in double primaries merely because of germline influences on expression. To evaluate this issue, we examined the correlations of expression levels in breast tumors and normal tissue using publically available data from The Cancer Genome Atlas 3. Of the 196 genes in our panel the mean normal-tumor correlation is a modestly positive 0.113. Importantly for our purposes the correlations in expression of double primaries do not seem to be strongly related to these tumor-normal correlations (correlation of the correlation coefficients is 0.05). For the key gene in our analyses, *ESR1*, the tumor normal correlation is 0.096 while the correlation between double primaries is 0.517, indicating only modest inflation. A fourth limitation is that our case–control analysis involved risk factors identified in the 1980s and these do not include important factors that have since been identified such as mammographic density 38 and germ-line mutations in various genes identified from family studies and genome-wide investigations 39.

In summary, our study has demonstrated the potential for studying etiologic heterogeneity using new methodology. The methods are aligned with the growing field of molecular pathologic epidemiology that seeks to understand the relationships of cancer risk factors with molecular characteristics of the tumors 40–42. The major results are consistent with a growing literature that confirms the etiological distinctiveness of breast cancers classified on the basis of expression of the estrogen receptor gene. The method seeks to identify the most etiologically distinctive subtypes, and in so doing to optimize risk prediction and the design of future epidemiological studies to identify new risk factors. Future studies of this type need larger sample sizes and more extensive genomic profiling of tumors in order to provide reliable and definitive evidence regarding etiologic heterogeneity.

## Supporting Information

Figure S1. Concordance of immunohistochemistry results and Nanostring results. **Table S1.** Full list of genes studied. **Table S2.** Results for all genes in the panel.
