# Supplementary material for: Identifying Etiologically Distinct Sub-Types of Cancer: A Demonstration Project Involving Breast Cancer
Source: Cancer Med. 2015 May 13;4(9):1432–9. doi: 10.1002/cam4.456 (PMC4567028; doi:10.1002/cam4.456)
Supplement: Figure S1. — Concordance of immunohistochemistry results and Nanostring results. Table S1. Full list of genes studied. Table S2. Results for all genes in the panel. [file cam40004-1432-sd1.docx]

eTable 1. Full List of Genes Studied

| *AKR1C4* | *CDKN1B* | *FANCE* | *HSD11B1* | *KRT86* | *PLAU* | *SERPINB5* | *UGT1A1* |
| --- | --- | --- | --- | --- | --- | --- | --- |
| *AKR1D1* | *CDKN2A* | *FANCF* | *HSD11B2* | *LCMT1* | *POLR2A* | *SERPINE1* | *UGT1A10* |
| *AR* | *CHEK1* | *FANCG* | *HSD17B1* | *LCMT2* | *PPARGC1A* | *SLC7A5* | *UGT1A3* |
| *ARSB* | *CHEK2* | *FAS* | *HSD17B12* | *MAP2K7* | *PPIA* | *SPEN* | *UGT1A4* |
| *ARSD* | *CLDN7* | *FASLG* | *HSD17B2* | *MAPK1* | *PPP1R15A* | *SPRR1B* | *UGT1A5* |
| *ARSE* | *CLU* | *FGF1* | *HSD17B3* | *MAPK3* | *PRMT2* | *SRA1* | *UGT1A6* |
| *ATF2* | *COL6A1* | *FHL5* | *HSD17B7* | *MED1* | *PRMT3* | *SRC* | *UGT1A7* |
| *ATM* | *CREBBP* | *FLRT1* | *HSD17B8* | *MEF2C* | *PRMT5* | *SRD5A1* | *UGT2A1* |
| *ATR* | *CTNNB1* | *FOSL1* | *HSD3B1* | *METTL2B* | *PRMT6* | *SRD5A2* | *UGT2A3* |
| *AZGP1* | *CTSB* | *GABRP* | *HSD3B2* | *METTL6* | *PRMT7* | *STC2* | *UGT2B10* |
| *BAD* | *CTSD* | *GATA3* | *HSPB1* | *MKI67* | *PRMT8* | *STS* | *UGT2B17* |
| *BAG1* | *CYP11B1* | *GNAS* | *HUS1* | *MRE11A* | *PTEN* | *SULT1E1* | *UGT2B7* |
| *BCL2* | *CYP11B2* | *GRIP1* | *ID2* | *MT3* | *PTGS2* | *SULT2A1* | *VEGFA* |
| *BCL2L2* | *CYP19A1* | *GSN* | *IGFBP2* | *MUC1* | *RAC2* | *SULT2B1* | *WBSCR22* |
| *BRCA1* | *DLC1* | *GTF2A1* | *IL2RA* | *NBN* | *RAD1* | *TBP* |  |
| *BRCA2* | *DST* | *GTF2E1* | *IL6* | *NCOR2* | *RAD17* | *TFF1* |  |
| *C3* | *EGFR* | *GTF2F1* | *IL6R* | *NFYB* | *RAD50* | *TFF3* |  |
| *CARM1* | *EP300* | *HDAC1* | *IL6ST* | *NGF* | *RAD51* | *TGFA* |  |
| *CCNA1* | *ERBB2* | *HDAC2* | *ITGA6* | *NGFR* | *RAD9A* | *THBS1* |  |
| *CCNA2* | *ERCC3* | *HDAC3* | *ITGB4* | *NME1* | *RPL13A* | *THBS2* |  |
| *CCND1* | *ESR1* | *HDAC4* | *JUN* | *NR0B1* | *RPL27* | *THBS4* | *CLTC** |
| *CCNE1* | *ESR2* | *HDAC5* | *KIT* | *NRIP1* | *S100A2* | *TIE1* | *GAPDH** |
| *CCNE2* | *F3* | *HDAC6* | *KLF5* | *PAPPA* | *SCGB1D2* | *TNFAIP2* | *GUSB** |
| *CD44* | *FANCA* | *HEMK1* | *KLK5* | *PELP1* | *SCGB2A1* | *TOP2A* | *HPRT1** |
| *CDH1* | *FANCC* | *HIST2H3C* | *KRT18* | *PGR* | *SCGB2A2* | *TP53* | *PGK1** |
| *CDKN1A* | *FANCD2* | *HMGB1* | *KRT19* | *PHB2* | *SERPINA3* | *TREX1* | *TUBB** |

eTable 2. Results for all Genes in the Panel

| Double Primary Analysis | | | | Case-Control Analysis | | |
| --- | --- | --- | --- | --- | --- | --- |
| Gene | OR | D* | p-value | Gene | D | p-value |
| *KRT19* | 13.8 | 0.66 | <0.001 | *GATA3* | 0.11 | <0.001 |
| *HSD17B1* | 8.2 | 0.52 | 0.002 | *ESR1* | 0.11 | <0.001 |
| *TOP2A* | 7.3 | 0.50 | 0.005 | *IL6ST* | 0.10 | <0.001 |
| *HPRT1* | 6.4 | 0.46 | 0.011 | *TFF1* | 0.10 | <0.001 |
| *IL6ST* | 6.4 | 0.46 | 0.006 | *BCL2* | 0.10 | <0.001 |
| *PRMT5* | 6.3 | 0.46 | 0.007 | *HMGB1* | 0.10 | <0.001 |
| *ID2* | 6.3 | 0.46 | 0.016 | *TFF3* | 0.09 | <0.001 |
| *MKI67* | 5.5 | 0.43 | 0.021 | *NRIP1* | 0.09 | <0.001 |
| *ATR* | 5.5 | 0.42 | 0.015 | *BCL2L2* | 0.09 | <0.001 |
| *ESR1* | 5.5 | 0.42 | 0.015 | *SULT2A1* | 0.09 | <0.001 |
| *PGR* | 5.5 | 0.42 | 0.015 | *PGR* | 0.08 | <0.001 |
| *AZGP1* | 5.4 | 0.42 | 0.014 | *GTF2F1* | 0.08 | 0.001 |
| *GAPDH* | 4.6 | 0.38 | 0.030 | *ERCC3* | 0.08 | 0.001 |
| *NME1* | 4.5 | 0.38 | 0.019 | *PGK1* | 0.08 | 0.001 |
| *MT3* | 4.4 | 0.37 | 0.032 | *NFYB* | 0.08 | 0.001 |
| *MUC1* | 4.4 | 0.37 | 0.032 | *JUN* | 0.08 | 0.001 |
| *HSPB1* | 4.4 | 0.37 | 0.040 | *SLC7A5* | 0.08 | 0.001 |
| *NRIP1* | 3.8 | 0.33 | 0.053 | *GABRP* | 0.08 | 0.001 |
| *SERPINA3* | 3.8 | 0.33 | 0.040 | *KRT19* | 0.08 | 0.001 |
| *RPL27* | 3.7 | 0.32 | 0.065 | *F3* | 0.08 | 0.002 |
| *CHEK1* | 3.6 | 0.32 | 0.063 | *RAD50* | 0.08 | 0.002 |
| *CDH1* | 3.4 | 0.31 | 0.066 | *ARSD* | 0.07 | 0.002 |
| *HSD17B7* | 3.4 | 0.31 | 0.066 | *CCND1* | 0.07 | 0.003 |
| *VEGFA* | 3.3 | 0.3 | 0.116 | *CREBBP* | 0.07 | 0.003 |
| *GUSB* | 3.2 | 0.29 | 0.076 | *MKI67* | 0.07 | 0.004 |
| *GATA3* | 3.1 | 0.28 | 0.124 | *HEMK1* | 0.07 | 0.005 |
| *F3* | 3.0 | 0.28 | 0.121 | *THBS4* | 0.07 | 0.006 |
| *PGK1* | 3.0 | 0.28 | 0.121 | *TUBB* | 0.07 | 0.006 |
| *AR* | 3.0 | 0.28 | 0.129 | *THBS2* | 0.07 | 0.007 |
| *FANCC* | 3.0 | 0.28 | 0.129 | *KRT18* | 0.07 | 0.008 |
| *PRMT8* | 3.0 | 0.28 | 0.129 | *AR* | 0.06 | 0.009 |
| *BCL2* | 3.0 | 0.27 | 0.127 | *CYP11B2* | 0.06 | 0.009 |
| *FAS* | 3.0 | 0.27 | 0.127 | *SCGB2A2* | 0.06 | 0.011 |
| *TFF3* | 3.0 | 0.27 | 0.127 | *CCNE1* | 0.06 | 0.012 |
| *THBS1* | 3.0 | 0.27 | 0.130 | *NBN* | 0.06 | 0.012 |
| *TUBB* | 2.9 | 0.27 | 0.125 | *MAPK3* | 0.06 | 0.013 |
| *CCNE1* | 2.9 | 0.27 | 0.125 | *SCGB1D2* | 0.06 | 0.014 |
| *GABRP* | 2.8 | 0.26 | 0.123 | *CCNA2* | 0.06 | 0.014 |
| *DST* | 2.8 | 0.25 | 0.203 | *FANCF* | 0.06 | 0.014 |
| *CCNA2* | 2.7 | 0.25 | 0.127 | *HSD3B2* | 0.06 | 0.014 |
| *ARSB* | 2.5 | 0.23 | 0.223 | *MUC1* | 0.06 | 0.016 |
| *HSD3B1* | 2.5 | 0.23 | 0.223 | *BRCA1* | 0.06 | 0.017 |
| *SLC7A5* | 2.5 | 0.23 | 0.223 | *HSPB1* | 0.06 | 0.018 |
| *ERBB2* | 2.5 | 0.23 | 0.227 | *PELP1* | 0.06 | 0.019 |
| *HEMK1* | 2.5 | 0.23 | 0.227 | *LCMT1* | 0.06 | 0.022 |
| *CDKN1B* | 2.5 | 0.23 | 0.220 | *SRD5A1* | 0.06 | 0.022 |
| *HDAC5* | 2.5 | 0.23 | 0.220 | *SCGB2A1* | 0.06 | 0.022 |
| *IGFBP2* | 2.5 | 0.23 | 0.220 | *HDAC3* | 0.06 | 0.025 |
| *PPIA* | 2.5 | 0.23 | 0.220 | *UGT1A3* | 0.06 | 0.026 |
| *IL2RA* | 2.4 | 0.22 | 0.225 | *HDAC2* | 0.06 | 0.027 |
| *LCMT1* | 2.4 | 0.22 | 0.210 | *UGT2B10* | 0.06 | 0.030 |
| *PPARGC1A* | 2.4 | 0.22 | 0.210 | *STC2* | 0.06 | 0.030 |
| *HSD11B2* | 2.4 | 0.22 | 0.223 | *RPL27* | 0.06 | 0.030 |
| *STC2* | 2.4 | 0.22 | 0.223 | *CLDN7* | 0.06 | 0.031 |
| *SULT2B1* | 2.4 | 0.22 | 0.223 | *SULT2B1* | 0.06 | 0.031 |
| *CLTC* | 2.4 | 0.22 | 0.218 | *CDKN1A* | 0.05 | 0.037 |
| *FASLG* | 2.3 | 0.21 | 0.222 | *TNFAIP2* | 0.05 | 0.037 |
| *CCNE2* | 2.3 | 0.21 | 0.313 | *IGFBP2* | 0.05 | 0.037 |
| *SERPINB5* | 2.3 | 0.2 | 0.315 | *RAD51* | 0.05 | 0.039 |
| *DLC1* | 2.2 | 0.2 | 0.228 | *FANCG* | 0.05 | 0.044 |
| *KRT18* | 2.2 | 0.2 | 0.225 | *RAD17* | 0.05 | 0.046 |
| *TGFA* | 2.2 | 0.19 | 0.238 | *CDKN1B* | 0.05 | 0.047 |
| *FOSL1* | 2.1 | 0.19 | 0.239 | *HSD17B3* | 0.05 | 0.047 |
| *CDKN1A* | 2.1 | 0.19 | 0.358 | *KLK5* | 0.05 | 0.048 |
| *SPRR1B* | 2.0 | 0.18 | 0.365 | *GAPDH* | 0.05 | 0.050 |
| *HDAC6* | 2.0 | 0.18 | 0.361 | *TOP2A* | 0.05 | 0.053 |
| *SERPINE1* | 2.0 | 0.18 | 0.361 | *PRMT7* | 0.05 | 0.054 |
| *PHB2* | 2.0 | 0.17 | 0.329 | *HSD17B12* | 0.05 | 0.060 |
| *GNAS* | 2.0 | 0.17 | 0.363 | *TREX1* | 0.05 | 0.060 |
| *CLU* | 2.0 | 0.17 | 0.359 | *HSD17B8* | 0.05 | 0.063 |
| *RAD9A* | 2.0 | 0.17 | 0.352 | *CARM1* | 0.05 | 0.064 |
| *CHEK2* | 1.9 | 0.16 | 0.340 | *PHB2* | 0.05 | 0.070 |
| *SRD5A1* | 1.8 | 0.15 | 0.522 | *SERPINB5* | 0.05 | 0.071 |
| *RAD17* | 1.8 | 0.15 | 0.372 | *UGT1A7* | 0.05 | 0.071 |
| *KIT* | 1.8 | 0.15 | 0.526 | *TBP* | 0.05 | 0.078 |
| *EGFR* | 1.8 | 0.15 | 0.373 | *SERPINA3* | 0.05 | 0.082 |
| *KRT86* | 1.8 | 0.15 | 0.373 | *ID2* | 0.05 | 0.084 |
| *THBS4* | 1.8 | 0.15 | 0.373 | *PRMT3* | 0.05 | 0.094 |
| *GTF2E1* | 1.8 | 0.14 | 0.537 | *CHEK1* | 0.05 | 0.095 |
| *SCGB1D2* | 1.8 | 0.14 | 0.537 | *CD44* | 0.05 | 0.096 |
| *BRCA2* | 1.7 | 0.14 | 0.382 | *TGFA* | 0.05 | 0.097 |
| *NCOR2* | 1.7 | 0.14 | 0.382 | *HDAC5* | 0.05 | 0.100 |
| *PTEN* | 1.7 | 0.14 | 0.382 | *PRMT5* | 0.05 | 0.104 |
| *AKR1D1* | 1.7 | 0.14 | 0.543 | *PRMT8* | 0.04 | 0.113 |
| *RAD50* | 1.7 | 0.14 | 0.543 | *GUSB* | 0.04 | 0.115 |
| *CYP11B1* | 1.7 | 0.13 | 0.547 | *MAPK1* | 0.04 | 0.115 |
| *CREBBP* | 1.7 | 0.13 | 0.545 | *HSD11B2* | 0.04 | 0.121 |
| *ESR2* | 1.7 | 0.13 | 0.545 | *METTL2B* | 0.04 | 0.133 |
| *FANCA* | 1.7 | 0.13 | 0.545 | *SPEN* | 0.04 | 0.137 |
| *NFYB* | 1.7 | 0.13 | 0.710 | *HIST2H3C* | 0.04 | 0.148 |
| *CD44* | 1.6 | 0.12 | 0.543 | *HSD17B2* | 0.04 | 0.149 |
| *CTSD* | 1.6 | 0.12 | 0.543 | *METTL6* | 0.04 | 0.154 |
| *HDAC1* | 1.6 | 0.12 | 0.543 | *GTF2A1* | 0.04 | 0.158 |
| *STS* | 1.6 | 0.12 | 0.543 | *SULT1E1* | 0.04 | 0.164 |
| *CARM1* | 1.6 | 0.12 | 0.531 | *PRMT6* | 0.04 | 0.168 |
| *ARSD* | 1.6 | 0.12 | 0.539 | *IL6* | 0.04 | 0.170 |
| *FANCE* | 1.6 | 0.12 | 0.539 | *SRA1* | 0.04 | 0.174 |
| *IL6* | 1.6 | 0.12 | 0.539 | *ATF2* | 0.04 | 0.175 |
| *TREX1* | 1.6 | 0.12 | 0.539 | *FGF1* | 0.04 | 0.182 |
| *UGT1A4* | 1.6 | 0.11 | 0.542 | *RAD9A* | 0.04 | 0.187 |
| *POLR2A* | 1.6 | 0.11 | 0.547 | *RPL13A* | 0.04 | 0.188 |
| *BCL2L2* | 1.5 | 0.1 | 0.545 | *ITGB4* | 0.04 | 0.189 |
| *ITGB4* | 1.5 | 0.1 | 0.545 | *FANCC* | 0.04 | 0.190 |
| *UGT1A7* | 1.5 | 0.1 | 0.557 | *LCMT2* | 0.04 | 0.200 |
| *NGF* | 1.4 | 0.09 | 0.563 | *IL2RA* | 0.04 | 0.203 |
| *UGT1A10* | 1.4 | 0.09 | 0.563 | *AZGP1* | 0.04 | 0.208 |
| *HSD17B8* | 1.4 | 0.08 | 0.761 | *AKR1D1* | 0.04 | 0.210 |
| *UGT2B17* | 1.4 | 0.08 | 0.761 | *PLAU* | 0.04 | 0.220 |
| *MAPK1* | 1.4 | 0.08 | 0.752 | *KIT* | 0.04 | 0.228 |
| *TIE1* | 1.4 | 0.08 | 0.752 | *STS* | 0.04 | 0.229 |
| *PTGS2* | 1.4 | 0.08 | 0.752 | *PTEN* | 0.04 | 0.230 |
| *FGF1* | 1.4 | 0.08 | 0.762 | *C3* | 0.04 | 0.237 |
| *TFF1* | 1.4 | 0.08 | 0.762 | *PAPPA* | 0.04 | 0.237 |
| *HDAC2* | 1.4 | 0.08 | 0.749 | *GRIP1* | 0.04 | 0.239 |
| *KLF5* | 1.4 | 0.08 | 0.76 | *ARSB* | 0.04 | 0.242 |
| *RPL13A* | 1.4 | 0.08 | 0.76 | *FHL5* | 0.04 | 0.247 |
| *SCGB2A1* | 1.4 | 0.08 | 0.76 | *CDKN2A* | 0.04 | 0.248 |
| *HIST2H3C* | 1.3 | 0.07 | 0.761 | *RAC2* | 0.04 | 0.248 |
| *UGT1A1* | 1.3 | 0.07 | 0.761 | *ERBB2* | 0.04 | 0.258 |
| *CTNNB1* | 1.3 | 0.07 | 0.757 | *MAP2K7* | 0.04 | 0.272 |
| *HUS1* | 1.3 | 0.07 | 0.76 | *UGT1A10* | 0.04 | 0.278 |
| *KLK5* | 1.3 | 0.07 | 0.752 | *KRT86* | 0.04 | 0.295 |
| *BRCA1* | 1.3 | 0.06 | 0.748 | *CTNNB1* | 0.03 | 0.313 |
| *WBSCR22* | 1.3 | 0.06 | 0.748 | *FLRT1* | 0.03 | 0.317 |
| *CDKN2A* | 1.3 | 0.06 | 0.76 | *KLF5* | 0.03 | 0.327 |
| *GTF2F1* | 1.3 | 0.06 | 0.761 | *NCOR2* | 0.03 | 0.327 |
| *HSD17B12* | 1.3 | 0.06 | 0.766 | *CYP19A1* | 0.03 | 0.328 |
| *AKR1C4* | 1.2 | 0.05 | 0.771 | *COL6A1* | 0.03 | 0.330 |
| *GTF2A1* | 1.2 | 0.05 | 1.000 | *CTSD* | 0.03 | 0.338 |
| *NGFR* | 1.2 | 0.05 | 1.000 | *HSD3B1* | 0.03 | 0.341 |
| *ATF2* | 1.2 | 0.05 | 1.000 | *UGT1A4* | 0.03 | 0.346 |
| *CCND1* | 1.2 | 0.05 | 1.000 | *HSD11B1* | 0.03 | 0.348 |
| *CTSB* | 1.2 | 0.05 | 1.000 | *UGT1A1* | 0.03 | 0.359 |
| *ITGA6* | 1.2 | 0.05 | 1.000 | *CYP11B1* | 0.03 | 0.374 |
| *UGT2B7* | 1.2 | 0.04 | 1.000 | *FANCD2* | 0.03 | 0.376 |
| *BAD* | 1.2 | 0.04 | 1.000 | *WBSCR22* | 0.03 | 0.385 |
| *HSD17B3* | 1.2 | 0.04 | 1.000 | *UGT2A1* | 0.03 | 0.386 |
| *PRMT6* | 1.2 | 0.04 | 1.000 | *THBS1* | 0.03 | 0.386 |
| *TNFAIP2* | 1.2 | 0.04 | 1.000 | *DST* | 0.03 | 0.393 |
| *MED1* | 1.2 | 0.04 | 1.000 | *CLU* | 0.03 | 0.401 |
| *RAD51* | 1.2 | 0.04 | 1.000 | *HDAC4* | 0.03 | 0.421 |
| *FANCD2* | 1.2 | 0.04 | 1.000 | *BRCA2* | 0.03 | 0.423 |
| *SULT2A1* | 1.2 | 0.03 | 1.000 | *MRE11A* | 0.03 | 0.425 |
| *HSD3B2* | 1.1 | 0.03 | 1.000 | *RAD1* | 0.03 | 0.435 |
| *PAPPA* | 1.1 | 0.03 | 1.000 | *UGT2B7* | 0.03 | 0.439 |
| *UGT1A6* | 1.1 | 0.02 | 1.000 | *UGT1A5* | 0.03 | 0.442 |
| *METTL2B* | 1.1 | 0.02 | 1.000 | *DLC1* | 0.03 | 0.45 |
| *TP53* | 1.1 | 0.02 | 1.000 | *TIE1* | 0.03 | 0.472 |
| *SRA1* | 1.0 | 0.01 | 1.000 | *ATM* | 0.03 | 0.487 |
| *TBP* | 1.0 | 0.01 | 1.000 | *FAS* | 0.03 | 0.488 |
| *HSD17B2* | 1.0 | 0.01 | 1.000 | *SPRR1B* | 0.03 | 0.504 |
| *C3* | 1.0 | 0.01 | 1.000 | *GTF2E1* | 0.03 | 0.504 |
| *BAG1* | 1.0 | 0 | 1.000 | *HPRT1* | 0.03 | 0.514 |
| *PRMT7* | 1.0 | 0 | 1.000 | *GNAS* | 0.03 | 0.520 |
| *UGT2A1* | 1.0 | 0 | 1.000 | *HSD17B1* | 0.03 | 0.524 |
| *UGT2A3* | 1.0 | 0 | 1.000 | *EP300* | 0.03 | 0.528 |
| *GRIP1* | 1.0 | 0 | 1.000 | *AKR1C4* | 0.03 | 0.538 |
| *NR0B1* | 1.0 | 0 | 1.000 | *ARSE* | 0.03 | 0.557 |
| *CYP19A1* | 1.0 | -0.01 | 1.000 | *IL6R* | 0.03 | 0.56 |
| *UGT1A5* | 1.0 | -0.01 | 1.000 | *FANCE* | 0.03 | 0.564 |
| *SRD5A2* | 1.0 | -0.01 | 1.000 | *UGT1A6* | 0.03 | 0.573 |
| *LCMT2* | 0.9 | -0.02 | 1.000 | *NR0B1* | 0.03 | 0.574 |
| *CYP11B2* | 0.9 | -0.02 | 1.000 | *PPP1R15A* | 0.03 | 0.581 |
| *ATM* | 0.9 | -0.03 | 1.000 | *HDAC1* | 0.03 | 0.589 |
| *MRE11A* | 0.9 | -0.03 | 1.000 | *HDAC6* | 0.03 | 0.596 |
| *S100A2* | 0.9 | -0.03 | 1.000 | *CTSB* | 0.03 | 0.603 |
| *CCNA1* | 0.8 | -0.04 | 1.000 | *BAG1* | 0.03 | 0.619 |
| *THBS2* | 0.8 | -0.04 | 1.000 | *UGT2B17* | 0.03 | 0.619 |
| *SCGB2A2* | 0.8 | -0.04 | 1.000 | *MEF2C* | 0.03 | 0.622 |
| *FANCF* | 0.8 | -0.05 | 1.000 | *HSD17B7* | 0.03 | 0.628 |
| *FLRT1* | 0.8 | -0.05 | 0.771 | *ATR* | 0.02 | 0.631 |
| *HMGB1* | 0.8 | -0.05 | 1.000 | *FANCA* | 0.02 | 0.636 |
| *MAPK3* | 0.8 | -0.05 | 0.759 | *PPIA* | 0.02 | 0.637 |
| *EP300* | 0.8 | -0.05 | 0.767 | *S100A2* | 0.02 | 0.640 |
| *NBN* | 0.8 | -0.05 | 0.767 | *ESR2* | 0.02 | 0.681 |
| *CLDN7* | 0.8 | -0.06 | 0.766 | *PRMT2* | 0.02 | 0.684 |
| *JUN* | 0.8 | -0.06 | 0.766 | *VEGFA* | 0.02 | 0.687 |
| *MAP2K7* | 0.7 | -0.07 | 0.760 | *ITGA6* | 0.02 | 0.697 |
| *FHL5* | 0.7 | -0.08 | 0.758 | *CCNE2* | 0.02 | 0.702 |
| *PRMT2* | 0.7 | -0.08 | 0.761 | *CDH1* | 0.02 | 0.703 |
| *HSD11B1* | 0.7 | -0.09 | 0.744 | *CLTC* | 0.02 | 0.707 |
| *PPP1R15A* | 0.7 | -0.09 | 0.763 | *MT3* | 0.02 | 0.775 |
| *IL6R* | 0.7 | -0.09 | 0.760 | *NGFR* | 0.02 | 0.776 |
| *FANCG* | 0.7 | -0.1 | 0.755 | *FASLG* | 0.02 | 0.778 |
| *GSN* | 0.7 | -0.1 | 0.755 | *PTGS2* | 0.02 | 0.781 |
| *SULT1E1* | 0.6 | -0.12 | 0.534 | *HUS1* | 0.02 | 0.786 |
| *SRC* | 0.6 | -0.13 | 0.535 | *CCNA1* | 0.02 | 0.789 |
| *METTL6* | 0.6 | -0.14 | 0.543 | *GSN* | 0.02 | 0.792 |
| *PELP1* | 0.6 | -0.14 | 0.382 | *TP53* | 0.02 | 0.800 |
| *MEF2C* | 0.5 | -0.17 | 0.359 | *EGFR* | 0.02 | 0.823 |
| *COL6A1* | 0.5 | -0.17 | 0.363 | *UGT2A3* | 0.02 | 0.828 |
| *HDAC3* | 0.5 | -0.17 | 0.363 | *SERPINE1* | 0.02 | 0.842 |
| *RAD1* | 0.5 | -0.18 | 0.365 | *SRC* | 0.02 | 0.848 |
| *ERCC3* | 0.5 | -0.18 | 0.364 | *SRD5A2* | 0.02 | 0.867 |
| *PRMT3* | 0.5 | -0.18 | 0.246 | *CHEK2* | 0.02 | 0.876 |
| *SPEN* | 0.5 | -0.19 | 0.358 | *MED1* | 0.02 | 0.899 |
| *RAC2* | 0.5 | -0.19 | 0.347 | *NGF* | 0.02 | 0.901 |
| *UGT2B10* | 0.4 | -0.22 | 0.223 | *POLR2A* | 0.02 | 0.918 |
| *HDAC4* | 0.4 | -0.23 | 0.211 | *BAD* | 0.01 | 0.930 |
| *UGT1A3* | 0.3 | -0.27 | 0.124 | *FOSL1* | 0.01 | 0.938 |
| *ARSE* | 0.3 | -0.28 | 0.129 | *PPARGC1A* | 0.01 | 0.955 |
| *PLAU* | 0.2 | -0.38 | 0.030 | *NME1* | 0.01 | 0.963 |

eFigure 1. Concordance of immunohistochemistry results and Nanostring results
